# Chronological Age and DNA Damage Accumulation in Blood Mononuclear Cells: A Linear Association in Healthy Humans after 50 Years of Age

**DOI:** 10.3390/ijms24087148

**Published:** 2023-04-12

**Authors:** Nikolaos I. Vlachogiannis, Panagiotis A. Ntouros, Maria Pappa, Evrydiki Kravvariti, Evangelia Georgia Kostaki, Georgios E. Fragoulis, Christina Papanikolaou, Dimitra Mavroeidi, Vasiliki-Kalliopi Bournia, Stylianos Panopoulos, Katerina Laskari, Aikaterini Arida, Vassilis G. Gorgoulis, Maria G. Tektonidou, Dimitrios Paraskevis, Petros P. Sfikakis, Vassilis L. Souliotis

**Affiliations:** 1 First Department of Propaedeutic Internal Medicine and Joint Rheumatology Program, National and Kapodistrian University of Athens Medical School, 115 27 Athens, Greece; 2 Postgraduate Medical Studies in Geriatric Syndromes and Physiology of Aging, National and Kapodistrian University of Athens Medical School, 115 27 Athens, Greece; 3 Department of Hygiene, Epidemiology and Medical Statistics, National and Kapodistrian University of Athens Medical School, 115 27 Athens, Greece; 4 Institute of Chemical Biology, National Hellenic Research Foundation, 116 35 Athens, Greece; 5 Molecular Carcinogenesis Group, Department of Histology and Embryology, National Kapodistrian University of Athens Medical School, 115 27 Athens, Greece

**Keywords:** chronological age, DNA damage response, Comet assay, oxidative stress, apurinic/apyrimidinic sites, endogenous DNA damage, double-strand breaks repair capacity

## Abstract

Aging is characterized by the progressive deregulation of homeostatic mechanisms causing the accumulation of macromolecular damage, including DNA damage, progressive decline in organ function and chronic diseases. Since several features of the aging phenotype are closely related to defects in the DNA damage response (DDR) network, we have herein investigated the relationship between chronological age and DDR signals in peripheral blood mononuclear cells (PBMCs) from healthy individuals. DDR-associated parameters, including endogenous DNA damage (single-strand breaks and double-strand breaks (DSBs) measured by the alkaline comet assay (Olive Tail Moment (OTM); DSBs-only by γH2AX immunofluorescence staining), DSBs repair capacity, oxidative stress, and apurinic/apyrimidinic sites were evaluated in PBMCs of 243 individuals aged 18–75 years, free of any major comorbidity. While OTM values showed marginal correlation with age until 50 years (r_s_ = 0.41, *p* = 0.11), a linear relationship was observed after 50 years (r = 0.95, *p* < 0.001). Moreover, individuals older than 50 years showed increased endogenous DSBs levels (γH2Ax), higher oxidative stress, augmented apurinic/apyrimidinic sites and decreased DSBs repair capacity than those with age lower than 50 years (all *p* < 0.001). Results were reproduced when we examined men and women separately. Prospective studies confirming the value of DNA damage accumulation as a biomarker of aging, as well as the presence of a relevant agethreshold, are warranted.

## 1. Introduction

Aging is a complex biological process characterized by the progressive deregulation of homeostatic mechanisms and reduced resilience to stress [1]. Multiple molecular and cellular alterations are associated with aging, which is related to most chronic disease states [2]. As Dr. Kirkwood has stated: “To understand the cell and molecular basis of aging is to unravel the multiplicity of mechanisms causing damage to accumulate and the complex array of systems working to keep damage at bay” [3]. While the biological basis of aging has not been elucidated to date, several features of the aging phenotype including genomic instability, mitochondrial dysfunction and cellular senescence are closely related to defects in the DNA damage response (DDR) network [4]. Indeed, defective DNA repair leads to the development of human progeroid syndromes characterized by an accelerated aging phenotype, suggesting that DNA damage accumulation accelerates physiological decline, thus contributing to the development of age-related diseases [4].

Whether the deregulation of DDR components and functionality can be employed as biomarkers of aging is debatable. DNA damage accumulation has been examined using the comet assay (single-cell gel electrophoresis), mainly in peripheral blood cells, in more than 13,000 research papers so far. Comet assay is a relatively easy, reproducible method which can be used with both freshlyisolated and properly cryopreserved samples [5,6], showing good correlation in 2 samples of the same individual collected even 1year apart [7]. Since the comet assay is characterized by its feasibility of application to a wide range of cells, including cells from tissues that are the prime targets for genotoxic insults, it is a well-accepted biomonitoring tool [8,9,10]. The alkaline version of comet assay, which measures both single-strand and double-strand DNA breaks (SSBs and DSBs, respectively) [5,6], has been used in most studies examining the potential relationship between age and endogenous DNA damage accumulation, as shown in a meta-analysis [11].

While many previous studies have examined the potential association between DNA damage accumulation measured by the comet assay and age, the results from different human studies are conflicting and show only modest associations between DNA damage accumulation in tissues and chronological age [11,12]. Technical limitations, such as the different conditions used in the quantification of DNA damage, or differences in the resilience of mechanisms related to less toxic DNA lesions, such as SSBs, as compared to the scarcer but highly toxic DSBs, may partly account for the conflicting results reported to date [11,12]. In our view, the major source of variability between the results of numerous such studies is the bias associated with the presence of multiple comorbidities, such as cancer, systemic autoimmune diseases, renal/heart disease, and even obesity, which are known to greatly increase the endogenous DNA damage levels [7,13,14]. Indeed, the lack of a systematic study ofa large number of individuals spanning all ages who are free of medical conditions that are known to impair DDR has prevented the description of a clear relationship between DNA damage and age per se.

Based on the above, we sought to examine the association(s) between chronological age and DDR-associated parameters in the absence of major comorbidities. Therefore, we have analyzed all relevant data derived from alkaline comet assay experiments on peripheral blood mononuclear cells (PBMCs) from a large number of apparently healthy individuals that have been conducted in our laboratory during the last decade. Moreover, since after mid-life a substantial decrease in physical and cognitive function is observed [15,16,17,18,19,20], we examined whether there is a chronological threshold after which deregulation of DDR is accelerated, thus probably reflecting the beginning of functional decline.

## 2. Results

### 2.1. Association between Age and DNA SSBs/DSBs Accumulation in PBMCs: The Critical Threshold of 50 Years

Both SSBs and DSBs of DNA were quantified by the alkaline comet assay [Olive tail moment (OTM) arbitrary units] in PBMCs derived from 246 apparently healthy humans (59% female) aged from 18 to 95 years. Individuals older than 75 years were excluded since only three observations were available for the age group between 76 and 95 years. The total number of observations after the exclusion of individuals aged more than 75 years (n = 3) was 243. The median age of individuals was 38 (IQR: 26, 59) years (Table 1).

**Figure 1:**
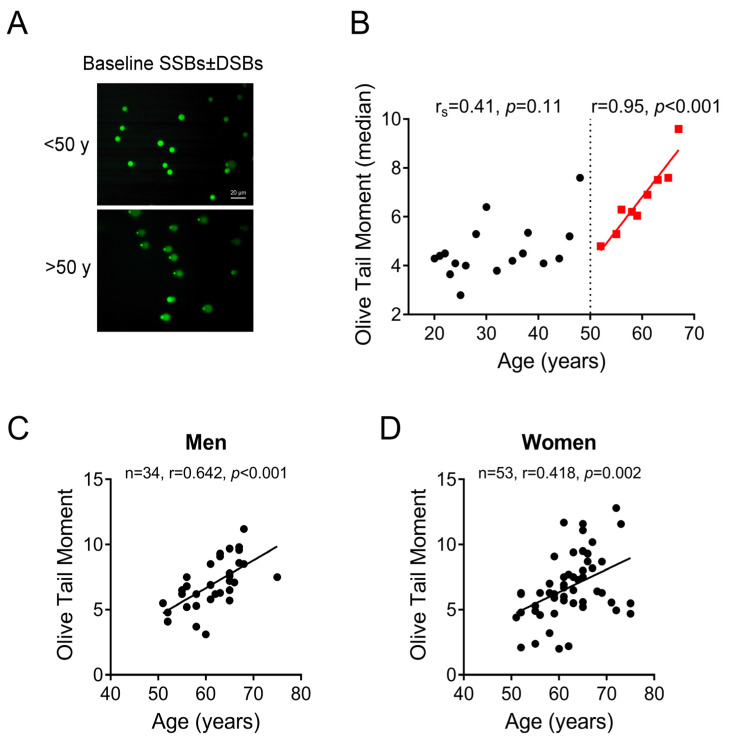
Endogenous SSBs ± DSBs in PBMCs. (**A**) Representative alkaline comet assay images of untreated PBMCs from a healthy individual under the age of 50 years and an individual over the age of 50 years (scale bar: 20 μm). (**B**) Correlation of DNA damage measured by alkaline comet assay with age in 243 apparently healthy humans (men and women). To reduce variance, we calculated the median OTM value per each age including only those ages with at least 4 observations. (**C**,**D**) Correlation of DNA damage measured by alkaline comet assay with age in 34 men older than 50 years (**C**), and in 53 women older than 50 years (**D**). Correlation coefficients were calculated using Pearson’s test for individuals aged ≥50 years and Spearman’s rank correlation coefficient (r_s_) for individuals younger than 50 years due to non-normal data distribution.

We found that OTM values differed up to 8-fold between participants (from 1.5 to 12.8) and the median (IQR) value was 5.2 (3.5, 6.94). To investigate the association of endogenous DNA SSBs/DSBs with age and in order to reduce variance, we calculated the median OTM value per each age including only those ages with at least 4 observations. OTM values showed a marginal correlation with age until the age of 50 years (r_s_ = 0.41, *p* = 0.11). In contrast, a strong positive correlation was observed between age and OTM in individuals older than 50 years (r = 0.95, *p* < 0.001) (Figure 1A,B). This correlationwas comparable when individual OTM values in men and women were examined separately (men: n = 34, r = 0.642; women: n = 53, r = 0.418) (Figure 1C,D). In view of these results, the study population was grouped into two categories: (i) individuals younger than 50 years (n = 156) and (ii) individuals 50 years and older (n = 87). The median age of those in the first age group was 28 (IQR: 24, 37) years and for those in the second one was 62 (IQR: 58, 65) years. Mann–Whitney U test showed that endogenous DNA SSBs/DSBs in the age group ≥50 years [median: 6.4 (IQR: 5.3, 8.5)] was higher than in the age group <50 years [median: 4.4 (IQR: 3.1, 6.0)] (*p* < 0.001). Furthermore, median regression analysis revealed that for the age group ≥50 years the predicted value of endogenous DNA SSBs/DSBs would be 2.02 (95% CI: 1.33–2.71; *p* < 0.001) units higher than that for the age group <50 years.

**Figure 2:**
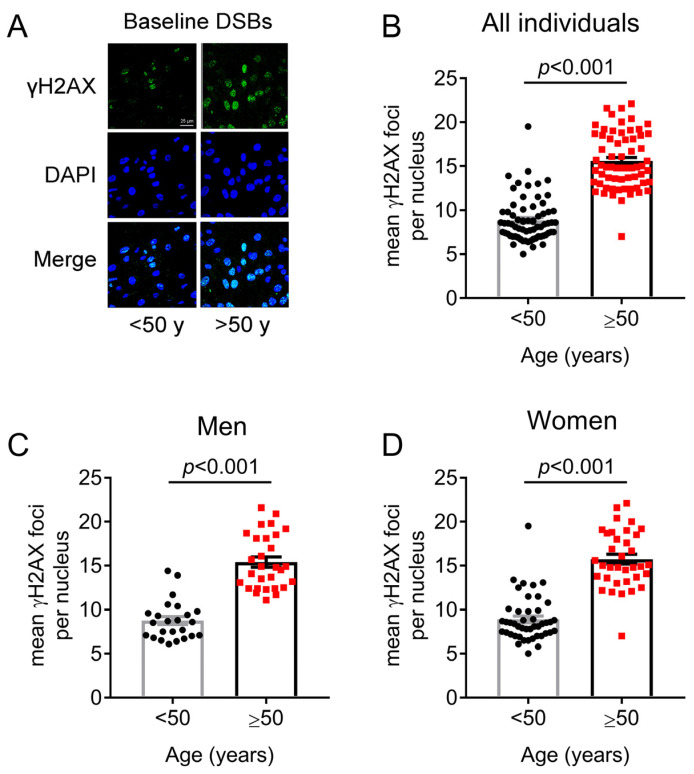
Endogenous DNA double-strand break levels in PBMCs. (**A**) Representative confocal microscopy images showing γH2AX staining from an individual under the age of 50 years (37 years old) and an individual over the age of 50 years (61 years old). Upper images, immunofluorescence γH2AX staining; middle, cell nuclei labeled with DAPI; bottom, merged (scale bar: 25 μm). (**B**–**D**) Endogenous DNA double-strand breaks assessed by γH2AX immunofluorescence staining in PBMCs of individuals younger or older than 50 years in the whole study population (mean ± SEM: <50 = 8.9 ± 0.3; ≥50 = 15.6 ± 0.4) (**B**), only in men (mean ± SEM: <50 = 8.8 ± 0.5; ≥50 = 15.4 ± 0.6) (**C**), and only in women (mean ± SEM: <50 = 8.9 ± 0.4; ≥50 = 15.8 ± 0.6) (**D**). The *p*-values are derived from Mann–Whitney U test. Bars and error bars represent mean and standard error of the mean (SEM).

In line with the previous findings, phosphorylated γH2AX levels, which are associated with the presence of DSBs [21,22], were also increased in individuals older than 50 years (Figure 2A,B), which held true for both men and women (Figure 2C,D), resembling the increase in SSBs/DSBs detected by the alkaline comet assay (Figure 1).

### 2.2. DNA Damage Formation and Repair Capacity of DNA Double Strand Breaks Differs before and after 50 Years of Age

**Figure 3:**
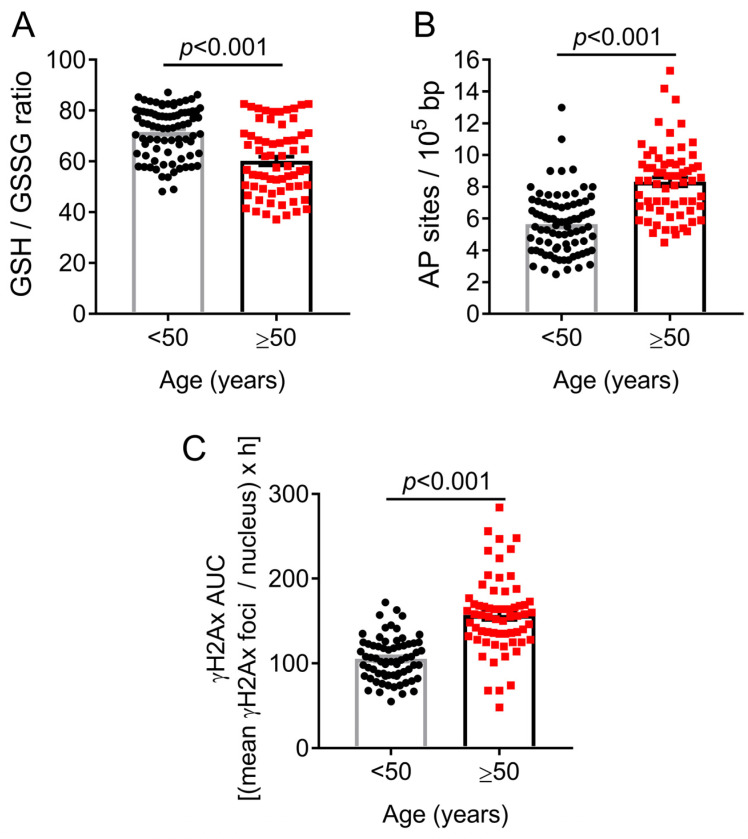
Oxidative stress, abasic sites and DSBs repair capacity in PBMCs. Bar graphs showing (**A**) the oxidative stress levels expressed as the ratio of reduced-to-oxidized glutathione (lower ratio is an indication of higher oxidative stress levels) (GSH/GSSG mean ± SEM: <50 = 71.6 ± 1.1; ≥50 = 60.2 ± 1.7), (**B**) the apurinic/apyrimidinic (AP) sites (AP sites/10^5^ nucleotides mean ± SEM: <50 = 5.7 ± 0.2; ≥50 = 8.3 ± 0.3) and (**C**) the melphalan-induced accumulation of γH2AX foci (expressed as AUC over 24 h incubation) in healthy individuals younger and older than 50 years (γH2AX AUC over 24 h mean ± SEM: <50 = 105.4 ± 3.1; ≥50 = 157.1 ± 5.6). The experiments shown were based on a minimum of 3 independent repeats. The *p*-values are derived from Mann–Whitney U test. Bars and error bars represent mean and standard error of the mean (SEM).

We next compared the levels of oxidative stress and abasic (apurinic/apyrimidinic) sites, which are indicative of DNA damage formation, as well as the kinetics of γH2AX (indicative of repair capacity of DSBs) after ex vivo treatment of PBMCs with melphalan, between healthy individuals younger and older than 50 years in available samples. We found that all parameters were significantly affected in older compared with younger individuals. Older individuals showed higher oxidative stress (as suggested by a lower reduced-to-oxidized glutathione ratio) (Figure 3A) and an increased number of apurinic/apyrimidinic sites (Figure 3B) (both *p* < 0.001). Moreover, the DSB repair capacity was measured as the Area under the Curve (AUC) of γH2AX foci over a 24 h period following treatment of cells with melphalan after extracting baseline γH2AX levels. Increased accumulation of melphalan-induced γH2AX over time, which is indicative of reduced DSBs repair capacity, was observed in older individuals (Figure 3C) (*p* < 0.001). Similar results were observed when we examined men and women separately (Figure S1).

## 3. Discussion

Herein we show that, in the absence of a known chronic (auto)immune-mediated, inflammatory or neoplastic disease, after the threshold of 50 years, age presents a strong linear relationship with DNA damage burden in human PBMCs measured by a standard method, namely the alkaline comet assay. This relationship is observed in both men and women. To the best of our knowledge, no previous study tested the hypothesis that a threshold for accelerated DNA damage accumulation in healthy humans exists.

Moreover, the toxic DSBs, estimated by γH2AX immunofluorescence, were also increased in men and women >50 years old compared to their younger counterparts. DSBs are among the most dangerous types of DNA damage, severely compromising the stability of the genome [14]. These lesions are repaired by two DNA repair mechanisms: homologous recombination (HR), a DNA repair pathway that acts in the S and G2 phases of the cell cycle, and non-homologous end joining (NHEJ), which is active throughout all phases of the cell cycle [23].

Markers indicative of DNA damage formation (oxidative stress and apurinic/apyrimidinic sites) were also significantly affected in both sexes after the age of 50, potentially accounting for the observed accumulation of DNA damage. The apurinic/apyrimidinic (AP) site is a common DNA lesion and an important intermediate in the DNA base excision repair (BER) pathway [24,25]. Several processes, including oxidative stress, alkylation of bases, and radiation, may lead to damaged DNA bases that are excised by DNA glycosylases resulting in the formation of apurinic/apyrimidinic sites [26]. Spontaneous cleavage of a glycosylic bond also results in the formation of AP sites [27]. Apurinic/apyrimidinic sites could be cytotoxic and mutagenic if they are not efficiently repaired [28,29]. In mammals, apurinic/apyrimidinic sites are removed by an AP endonuclease which incises the 5′ end of AP sites and results in the formation of a 5′-deoxyribose phosphate terminus that is repaired by the DNA polymerase beta [25].

Apart from increased DNA damage formation, individuals older than 50 years also showed decreased repairing capacity of melphalan-induced DSBs. Melphalan is a nitrogen mustard used to treat several types of cancer [30] which reacts with DNA to produce mainly N-alkylpurine-monoadducts, a small part of which forms interstrand crosslink (ICL), a highly cytotoxic DNA lesion [31]. N-alkylpurine-monoadducts are repaired through the mechanism of nucleotide excision repair (NER), whereas homologous recombination (HR), NER, and translesion synthesis are all required for ICL repair [32].In particular, ICL repair proceeds through the formation of DSBs, the most dangerous form of DNA damage [33].

Previous reviews and meta-analyses examining the relationship between DNA damage accumulation in peripheral blood leukocytes and age have reported little or no effect [11,12,34]. In contrast to our findings, a meta-analysis of 105 studies including 13,553 subjects—albeit with the majority having various comorbidities—reported only a smallvariation of endogenous DNA damage with increasing age [12]. Similarly, a review of 25 studies including individuals with more than 30 years of difference between the youngest and oldest participants, showed a positive association between age and DNA strand breaks in half, and no association in the remaining studies [34]. Of note, most studies used age strata instead of continuous data. Linear regression analysis including a total of 2879 individuals showed a 1.0% increase in DNA strand breaks per 1-year of increasing age [34]. Another study examining the association between age and DNA damage levels in PBMCs of individuals aged between 40 and 77 years also showed only a modest association [35]. More specifically, the authors examined the levels of DNA SSBs and DSBs, as well as the repair capacity of DSBs, in PBMCs of 216 individuals from a population-based sample of twins aged 40–77 years. They found no effect of sex in any measurement. Age had no effect on SSB levels or their repair, while DSB repair capacity tended to decrease with increasing age. Nevertheless, this study did not exclude individuals in poor health or with cancer [35], which greatly affect endogenous DNA damage levels.

Overall, the different comet assay descriptors used in various studies (e.g., % tail DNA vs.Olive tail moment), as well as the different conditions (e.g., different lysis conditions, different electrophoresis time) make it difficult to compare results between different studies [36]. Regarding the different comet assay descriptors, primary comet assay measurements include tail length, tail DNA, and DNA distribution profile in the tail, which are obtained by the fluorescent densitometric profiles of the comets [6,36]. All other measurements, such as Tail Moment, OTM, and % tail DNA are derived from the 3 primary measurements. While comet assay has been used in more than 13,000 published studies to date, including biomonitoring studies, there is no consensus on which comet descriptor reflects more precisely the DNA damage extent. The two most widely used descriptors to date include tail intensity (% tail DNA) and OTM. Tail intensity is expressed as % of total DNA fluorescence in the tail of the comet. OTM is calculated as the product of the tail length and the fraction of total DNA in the comet tail [5]. Although tail intensity is recommended as the best descriptor for DNA break frequencies since it uses a quantitative measure of damage (from 0 to 100%) [36], herein we opted to use OTM in order to compare with published results since this parameter has been used by most previous studies reporting on the association between DNA damage and age and/or comorbidities. It is well-established that the tail moment calculated by Olive et al. (Olive Tail Moment; OTM) [37] is particularly useful in describing heterogeneity within a cell population as it can pick up variations in DNA distribution within the tail. Moreover, tail extent moment and OTM have been found to be highly correlated with % tail DNA (*p* < 0.01) [38], which also holds true in our samples (r = 0.78 for OTM with % tail DNA).

The presence of comorbidities is, in our opinion, the most important confounding factor that may have hindered previous studies from revealing a relationship between DNA damage levels and age. Notably, when we performed an additional analysis in a second cohort comprising 234 patients (68% female) with chronic immune-mediated rheumatic diseases known to promote accumulation of DNA damage in peripheral blood mononuclear cells (systemic lupus erythematosus, rheumatoid arthritis, systemic sclerosis, Behcet’s disease, and primary antiphospholipid syndrome) [14,39,40,41,42,43,44], no correlation at any level was observed between chronological age and endogenous DNA SSBs/DSBs. Along this line, it seems that chronological age is no longer the primary source of DNA damage accumulation in the presence of persisting inflammation, which results in inflammaging and thus increased biological age [45,46]. In line with this notion, chronic inflammation was recently added to the hallmarks of aging [47].

The concept of an agethreshold around mid-life, after which functional decline is accelerated, is also supported by a large study examining grip strength across the life course combining 60,803 observations from 49,964 participants [15] which showed the gradual decline of grip strength after mid-life and accelerating deterioration with increasing age [15]. Moreover, the largest population-based study to date examining the relationship between DNA damage and age in PBMCs derived from 993 individuals aged 18–93 years showed that oxidative DNA damage was increased in women older than 50 years as compared to the women in the youngest age group (18–29 years old), although no association was observed between age and DNA strand breaks [48].

Since unrepaired DNA damage in humans may lead to functional defects or cell death, it is reasonable to hypothesize that a decline in DDR is related to aging. Indeed, when we examined the repair efficiency of the toxic DSBs, which are key DNA lesions affecting cell viability and longevity [49], we found that this mechanism is severely affected in individuals >50 years old. In line with this, multiple previous studies have reported a decrease in DNA repair mechanisms with increasing age. Levels of key DSBs repair enzymes, such as Ku70 and Mre11, were shown to decrease with increasing age in a study of CD4^+^ T cells from 48 subjects aged 20–80 years [50]. Beyond decreased levels, DDR proteins may be mis-localized in the cell, failing to reach the sites of DNA damage. For example, in a study including 24 individuals aged 20–89 years, PBMCs from elderly subjects showed lower nuclear localization and DNA binding of the Ku70/80 complex upon X-ray-irradiation [51]. Other studies have also reported the failure of the cellular machinery to detect DNA damage sites and signal their presence to initiate the appropriate repair processes (e.g., defective γH2AX response) [35]. Therefore, taken together, our results and previous reports suggest an age-related decline in the detection, signaling, and repair of DNA damage, partially due to mis-localization or decreased expression of key DDR components observed in aged individuals. Whether these changes are an effect or the cause of aging is the scope of future functional and prospective epidemiological studies.

It is generally accepted that high levels of endogenous DNA damage pose a serious threat to cellular health, as they may result in mutagenesis and genomic instability. Indeed, a growing number of reports have shown that increased DNA damage levels are associated with a wide range of clinical conditions such as cancer, autoimmune diseases, coronary artery disease, kidney disease, chronic obstructive pulmonary disease, multiple sclerosis, and Alzheimer’s disease [52,53,54,55]. Importantly, Vodicka et al. reviewed the DNA damage and repair capacity for 17 types of cancer measured by the comet assay and shed light on the utility of this method in evaluating cancer aetiology, disease prognosis, and treatment prediction [56]. Moreover, Bonassi et al. reported that increased DNA damage measured by the comet assay represents a crucial factor resulting in chronic diseases and eventually in death, and that measurement of DNA damage in circulating leukocytes may predict mortality risk [57].

Limitations of our study include an absence of data on smoking status, alcohol consumption and the exact body mass index (BMI), although patients with clinician-assessed morbid obesity, i.e., BMI > 35 kg/m^2^, were excluded. An additional limitation is that seasonal variation of OTM was not considered; indeed, previous studies have shown an impact of seasonal variations on the comet assay parameters in human biomonitoring, with higher baseline DNA damage levels being observed in the summer compared with all the other seasons [58]. Moreover, our study is retrospective but, to the best of our knowledge, no prospective study has been performed so far. Also, we did not perform a batch correction of our results, i.e., adjusting the value of each sample according to an internal standard, which seemed to attenuate the statistical significance of the results of previous studies [35]; but we included a positive control (PBMCs treated with 100 μM H_2_O_2_ for 30 min) in each comet assay to ensure cell lysis and electrophoresis worked properly, while all experiments were performed in the same laboratory. Additionally, although the levels of γH2AX foci per cell presented herein (mean ± SEM: <50 years = 8.9 ± 0.3; ≥50 years = 15.6 ± 0.4) are in accordance with our previous reports measuring γH2AX foci in PBMCs from healthy controls [42,59,60], they are higher than those reported by other studies. Indeed, Schurman and colleagues reported 4.25 ± 0.28γH2AX foci/cell [61] while other studies reported even lower levels [62]. This discrepancy can mostly be explained by the counting method. For example, a complication of the task of quantifying the levels of γH2AX could be related to the size of the foci measured since foci can be very small, occupying thousands of bp or very large, occupying 30 Mbp or more [63]. A further complication is that foci can occupy many levels along the vertical axis of the nucleus, thus making measurement difficult. Finally, only a limited number of studies have tested the differences between H2AX phosphorylation in fresh and cryopreserved PBMCs; among these, Sánchez-Flores and colleagues found a slight increase of H2AX phosphorylation in cryopreserved PBMCs than in fresh cells [64].

To conclude, our findings show that, in the absence of a known chronic inflammatory or neoplastic disease, after the 5th decade of life, the chronological age strongly correlates with DNA damage burden in human PBMCs. This correlation, taken together with increased oxidative stress and a decline of DNA repair mechanisms in apparently healthy individuals older than 50 years, suggests a progressive linear decline of the effective DNA damage response after middle age. Prospective studies not yet performed on whether these findings can be exploited as novel therapeutic targets and biomarkers of aging are warranted.

## 4. Materials and Methods

### 4.1. Study Cohort

A total of 246 apparently healthy individuals were included in our study. All study participants were free of major comorbidities including history of any type of malignancy, heart or renal disease, systemic autoimmune disease, diabetes mellitus, BMI > 35 kg/m^2^(clinician-assessed), as well as recent (last 2 weeks) infection or hospitalization. Moreover, older individuals who participated in this study had a clinical frailty scale score (CFS) < 4 since frailty has been related to increased DNA damage in PBMCs [59]. The study was approved by the “Laiko” Hospital Ethics Committee (Protocol Nr 1110 and 1348) and all participants provided written informed consent according to the declaration of Helsinki.

### 4.2. Peripheral Blood Mononuclear Cell Isolation

PBMCs were isolated from freshly drawn peripheral blood and purified using the standard Ficoll gradient centrifugation (Ficoll-Paque Plus, Sigma Aldrich, St. Louis, MO, USA; #GE17-1440-03) as described [59]. Cells were resuspended in freezing medium (90% fetal bovine serum, 10% dimethyl sulfoxide) and stored at −80 °C with gradual freezing until further processing.

### 4.3. Alkaline Comet Assay

Endogenous DNA damage accumulation was quantified by single-cell gel electrophoresis under alkaline conditions (alkaline comet assay) measuring SSBs and/or DSBs in DNA [5]. Detailed relevant information can be found in [59]. PBMCs were briefly resuspened at single-cell in low-point-melting agarose (1%) and loaded on slides precoated with 1% standard agarose and covered with coverslip. Slides were then left to dry at 4 °C for 30 min. Next, slides were covered in an alkaline lysis buffer (NaCl 2.5 M, EDTA 0.1 M, Tris 0.01 M; pH = 10, with the fresh addition of 1% Triton X-100) for 2 h at 4 °C. Next, we allowed the slides to equilibrate for 40 min in electrophoresis buffer and then performed electrophoresis of the samples for 30 min at 4 °C with 1 V/cm electrophoretic field strength (Electrophoresis buffer: 0.3M NaOH, 1mM EDTA). Finally, a neutralizing buffer (0.4 M Tris; pH = 7.5) was added on the slides for 10 min at 4 °C. Slides were finally washed with double-distilled H_2_O and left to dry overnight before staining with SYBR Gold Nucleic Acid Gel Stain (Thermo Fisher Scientific, Waltham, MA, USA, #S11494) and studied with a fluorescence microscope (Zeiss Axiophot, Oberkochen, Germany). A positive assay control (PBMCs treated with 100 μM H_2_O_2_ for 30 min) was included in each comet assay to ensure cell lysis and electrophoresis worked properly. We report the results of the Olive Tail Moment (OTM) [65], assessed by the ImageJ Analysis/Open Comet v1.3.1, an open-source software tool providing automated analysis of comet assay images. For each sample, 2 gels were scored and the average OTM value of 200 cells was calculated.

### 4.4. Immunofluorescence Detection of γH2AX Foci

Immunofluorescence antigen staining and confocal laser scanning microscopy for the analysis of γH2AX foci (H2AX phosphorylated at Ser139; #9718T, Cell Signaling Technology, Danvers, MA, USA) was performed as previouslydescribed [39]. Following the detection of a DSB, the histone H2A variant H2AX is quickly phosphorylated at Ser139 to create γH2AX [66], which is the first step in recruiting and localizing DNA repair proteins [21]. Therefore, the presence of γH2AX is commonly used as a marker of DSBs in human population studies [22]. Unlike comet assay methodology, standardized protocols for γH2AX foci assessment are lacking [64]. In our results, we present numbers of γH2AX foci per nucleus; this is considered the most rigorous approach compared to others, namely total nuclear fluorescence and scoring foci positivity [67]. The γH2AX foci per nucleus were manually counted in 200 cells per sample since this method allows a critical evaluation between unspecific signals and induced foci. Only foci within nucleus stained by DAPI were counted, while damaged nuclei and apoptotic cells were excluded. Similarly trained investigators were blinded to the participants’ age and gender during the analysis.

### 4.5. Assessment of DNA Damage Formation

To examine markers of DNA damage formation, oxidative stress was quantified using a luminescence-based system that measures the reduced glutathione (GSH) to oxidized glutathione (GSSG) ratio (GSH/GSSG) according to the manufacturer’s experimental protocol (GSH/GSSG-Glo™ Assay; Promega, #V6612, Madison, WI, USA). The levels of abasic (apurinic/apyrimidinic; AP) sites were also evaluated using the OxiSelect Oxidative DNA Damage Quantitation Kit (Cell Biolabs, Inc., San Diego, CA, USA, #STA-324) according to the manufacturer’s experimental protocol.

### 4.6. Repair Capacity of DNA DSBs

Finally, to examine the repair capacity of the DNA DSBs, freshly isolated PBMCs were treated with 100 μg/mL melphalan for 5 min at 37 °C in RPMI medium supplemented with 10% fetal bovine serum, 100 units/mL penicillin, 100 μg/mL streptomycin, and 2 mmol/L L-glutamine. Then, they were incubated in a drug-free medium for 0, 8 and 24 h, adhered to a coverslip, fixed, and stored at −80 °C until γH2AX analysis. The DSBs repair capacity was measured as the Area under the Curve (AUC) of γH2AX foci during the whole experiment (0–24 h) after extracting baseline γH2AX levels, as previously described [40,42].

### 4.7. Statistical Analysis

Data were summarized using medians and interquartile ranges (IQRs). Normality was tested with the Shapiro–Wilk test. Pairwise comparisons were performed using independent samples t-test or Mann–Whiney U test when the assumption of normality was violated. Correlations were examined with the use of Pearson’s correlation coefficientor Spearman’s rank test when data did not follow normal distribution. A quantile (median) regression model was fitted on 243 observations with endogenous DNA SSBs/DSBs as the outcome variable and age as a possible explanatory variable. *P*-values less than 0.05 were considered statistically significant. Statistical analyses were performed using Stata 16.0-StataCorp LLC software, SPSS v.26 (IBM, Armonk, NY, USA) and GraphPad PRISM 7 software was used to create graphs.

## Figures and Tables

**Table 1:** Age and sex distribution of the study participants (n = 243).

Age Group	Male No.	Female No.
18–29	36 (43.9)	46 (56.1)
30–49	29 (39.2)	45 (60.8)
50–69	33 (41.3)	47 (58.8)
70–75	1 (14.3)	6 (85.7)
Sum	99 (40.7)	144 (59.3)

## Data Availability

Data are contained within the article.

## Supplementary Materials

The following supporting information can be downloaded at: https://www.mdpi.com/article/10.3390/ijms24087148/s1.
