# Supplementary material for: Chronological Age and DNA Damage Accumulation in Blood Mononuclear Cells: A Linear Association in Healthy Humans after 50 Years of Age
Source: Int J Mol Sci. 2023 Apr 12;24(8):7148. doi: 10.3390/ijms24087148 (PMC10138488; doi:10.3390/ijms24087148)
Supplement: Supplementary file 1 [file ijms-24-07148-s001.zip › ijms-2252009-supplementary.pdf]

Supplementary Material for

Chronological age and DNA damage accumulation in blood mononuclear cells: a linear association in healthy humans after 50 years of age

Nikolaos I. Vlachogiannis [1,†], Panagiotis A. Ntouros [1], Maria Pappa [1,†], Evrydiki Kravvariti [1,2], Evangelia Georgia Kostaki [3], Georgios E. Fragoulis [1], Christina Papanikolaou [4], Dimitra Mavroeidi [4], Vasiliki-Kalliopi Bournia [1], Stylianos Panopoulos [1], Katerina Laskari [1], Aikaterini Arida [1], Vassilis G. Gorgoulis [5], Maria G. Tektonidou [1], Dimitrios Paraskevis [3], Petros P. Sfikakis [1,2], Vassilis L. Souliotis [1,4,*]

[1] First Department of Propaedeutic Internal Medicine and Joint Rheumatology Program, National and Kapodistrian University of Athens Medical School, 115 27 Athens, Greece

[2] Postgraduate Medical Studies in Geriatric Syndromes and Physiology of Aging, School of Medicine, NKUA, Athens, Greece

[3] Department of Hygiene, Epidemiology and Medical Statistics, National and Kapodistrian University of Athens Medical School, 115 27 Athens, Greece

[4] Institute of Chemical Biology, National Hellenic Research Foundation, 116 35 Athens, Greece

[5] Molecular Carcinogenesis Group, Department of Histology and Embryology, National Kapodistrian University of Athens Medical School, 115 27 Athens, Greece

* Correspondence: vls@eie.gr

† These authors contributed equally to this work

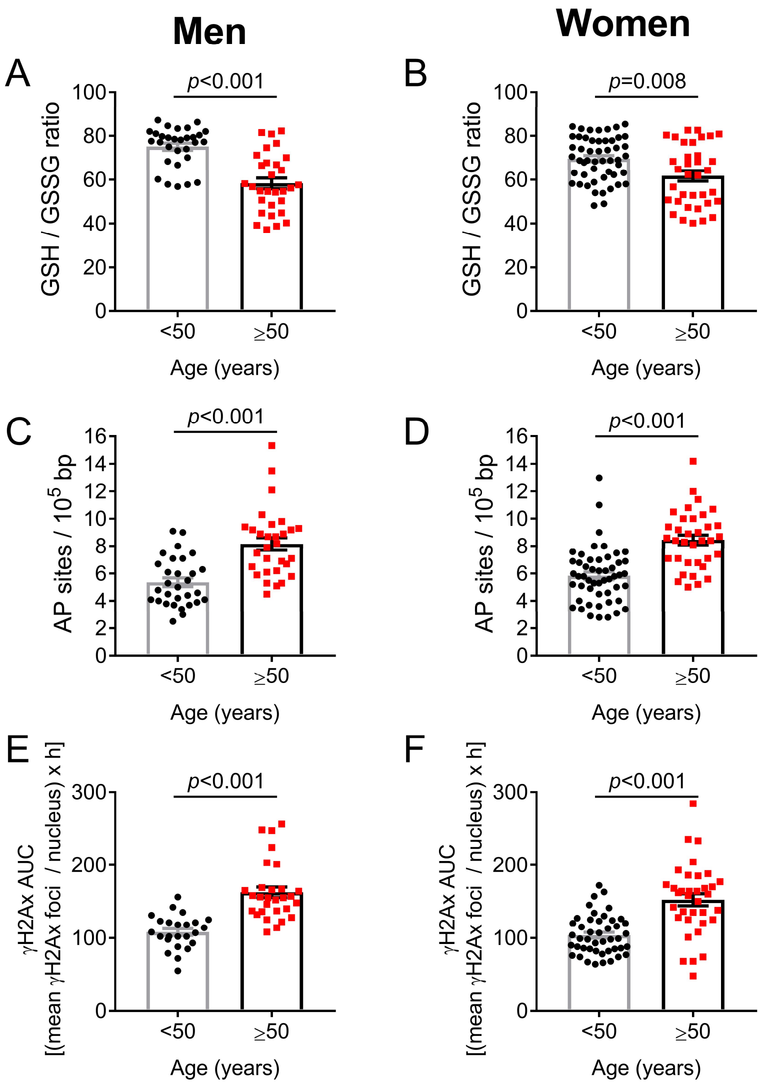

Figure S1. Oxidative stress, abasic sites and DSB repair capacity. Bar graphs showing (A,B) the oxidative stress levels expressed as the ratio of reduced to oxidized glutathione (lower ratio is an indication of higher oxidative stress levels) (GSH/GSSG mean±SEM, men: <50 = 75.1±1.6; ≥50 = 58.4±2.4; women: <50 = 69.6±1.4; ≥50 = 61.8±2.4), (C,D) the apurinic / apyrimidinic (AP) sites (AP sites per 10^5 nucleotides mean±SEM, men: <50 = 5.4±0.3; ≥50 = 8.2±0.5; women: <50 = 5.8±0.3; ≥50 = 8.5±0.4) and (E,F) the melphalan-induced accumulation of γH2AX foci (expressed as AUC over a 24 h period) (γH2AX AUC over 24h mean±SEM, men: <50 = 108.2±4.7; ≥50 = 162.6±7.4; women: <50 = 103.9±4.0; ≥50 = 152.5±8.3) in healthy men (A,C,E) and women (B,D,F) younger and older than 50 years. The experiments shown were based on a minimum of 3 independent repeats. *P*-values were derived from Mann-Whitney U test. Bars and error bars represent mean and standard error of the mean (SEM).
